# Surgery followed by adjuvant radiotherapy versus definitive radiotherapy for locally advanced squamous cell carcinoma of the pharynx and larynx: a propensity score-matched analysis

**DOI:** 10.1016/j.ctro.2026.101288

**Published:** 2026-09-10

**Authors:** Linda Agolli, Elidon Mici, Christine Langer, Gabriele A. Krombach, Sebastian Harth, Leon Wendrich, Stefan Gattenlöhner, Christoph Arens, Daniel Habermehl

**Affiliations:** a Department of Radiation Oncology, Justus-Liebig-University Giessen, Giessen University Hospital, Giessen, Germany; b Department of Oral and Maxillofacial Surgery, Trauma Hospital, BG Clinic, Berlin, Germany; c Department of Otorhinolaryngology, Justus-Liebig-University Giessen, Giessen University Hospital, Giessen, Germany; d Department of interventional Radiology and Diagnostic, Justus-Liebig-University Giessen, Giessen University Hospital, Giessen, Germany; e Institute of Pathology, Justus-Liebig-University Giessen, Giessen University Hospital, Giessen, Germany

**Keywords:** Head and neck cancer, Propensity score, Definitive radiotherapy, Adjuvant radiotherapy, Outcome

## Abstract

**Aim**: Locally advanced squamous cell carcinoma (SCC) of the pharynx and larynx is commonly treated with either primary surgery followed by adjuvant radiotherapy (RT) or definitive RT with or without concurrent systemic therapy. Comparative outcome data between these treatment strategies remain limited because retrospective analyses are frequently affected by treatment-selection bias. This study compared oncologic outcomes between primary surgery followed by adjuvant RT and definitive RT using propensity score matching to reduce baseline confounding.

**Methods**: A retrospective analysis was performed on 309 patients with locally advanced SCC of the oropharynx, hypopharynx, or larynx treated at our center. Propensity scores were generated using a multivariable logistic regression model including T stage, N stage, and age at diagnosis. One-to-one nearest-neighbor matching with a caliper of 2% resulted in 61 matched patient pairs treated with primary surgery followed by adjuvant RT or definitive RT. Survival outcomes were analyzed using the Kaplan–Meier method and compared using log-rank tests. Multivariable Cox regression analyses were performed to account for imbalances in previous malignancies after matching.

**Results**: After propensity score matching, 61 patients treated with primary surgery followed by adjuvant RT and 61 patients treated with definitive RT were included, with improved balanced baseline characteristics for the matching variables. Median OS was 81 months in the surgery followed by adjuvant RT group and was not reached in the definitive RT group, with no statistically significant difference between groups (*p* = 0.70). Numerical differences favored adjuvant RT for progression-free survival (median PFS: 81.0 vs. 43.4 months; *p* = 0.09) and loco-regional recurrence-free survival (*p* = 0.08), although these differences were not statistically significant. Lymphedema was the only toxicity significantly more frequent in the adjuvant RT cohort (33% vs. 13%, *p* = 0.017), while rates of other treatment-related toxicities were comparable. Multivariable analyses adjusted for previous malignancies confirmed the absence of significant associations between treatment modality and survivals.

**Conclusion**: Primary surgery followed by adjuvant RT and definitive RT achieved comparable overall survival in patients with locally advanced SCC of the pharynx and larynx after propensity score matching. However, the surgery plus adjuvant RT strategy demonstrated trends toward improved disease control and local control. These findings support individualized multidisciplinary treatment selection while prospective comparative studies remain warranted.

## Highlights

• Propensity score matching improved baseline comparability between adjuvant and definitive radiotherapy cohorts.
• Overall survival was comparable between primary surgery plus adjuvant RT and definitive RT.
• PFS and locoregional control numerically favored primary surgery plus adjuvant RT.
• Treatment approach was not independently associated with OS, PFS, or locoregional control.

## 1 Introduction

Locally advanced squamous cell carcinoma of the head and neck (LA-HNSCC) remains a major global health burden and accounts for substantial cancer-related morbidity and mortality worldwide [1]. Despite improvements in diagnostics, radiation techniques, systemic therapy, and surgical approaches, treatment of locally advanced disease continues to present considerable clinical challenges. Multimodal treatment strategies are generally required to achieve durable loco-regional control and long-term survival [2].

Current treatment strategies for LA-HNSCC generally consist either of definitive radiotherapy (RT), often combined with concurrent systemic therapy, or of primary surgical resection followed by adjuvant radiotherapy with or without chemotherapy. Both approaches are widely accepted and have demonstrated efficacy regarding disease control and survival outcomes [3]. Nevertheless, the optimal treatment strategy for individual patients remains controversial, particularly because each modality is associated with distinct advantages and treatment-related morbidity [4].

Definitive chemoradiation offers the potential benefit of organ preservation and avoidance of surgical morbidity, making it an attractive approach for many patients with advanced disease. In contrast, surgery followed by adjuvant radiotherapy may improve local disease control, especially in patients with high-risk pathological features such as advanced nodal involvement [2], [5].

However, direct comparisons between these strategies are complicated by substantial baseline differences between patient populations selected for surgery and those treated with definitive RT [5].

Retrospective studies are particularly susceptible to treatment allocation bias, as patients undergoing surgery often differ regarding tumor stage, nodal burden, age, and performance status [6].

In recent years, propensity score matching has become an important method for reducing baseline imbalances in observational oncologic studies and improving comparability between non-randomized treatment groups [7]. This approach is particularly relevant in head and neck oncology, where randomized comparisons between surgery-based and definitive RT strategies remain limited [8].

The present study is based on the same institutional database that has previously been used to investigate recurrence patterns and sex-related differences in locally advanced pharyngeal and laryngeal SCC [2], [4]. In contrast, the aim of the present study was therefore to compare survival outcomes, treatment-related toxicities, and recurrence patterns between patients receiving surgery followed by adjuvant RT and definitive RT in patients with LA-SCC of the pharynx and larynx using a propensity score–matched analysis.

## 2 Materials and methods

### 2.1 Patients and treatment

A retrospective review was performed regarding patients with SCC of the oropharynx, hypopharynx, or larynx who were treated at our Department of Radiation Oncology between 2016 and 2023. The study was approved by the local Ethics Committee, which waived the requirement for informed consent due to the retrospective study design. Eligible patients had histologically confirmed SCC and received either definitive RT or surgery followed by adjuvant RT with curative intent combined or not to systemic therapy. Inclusion criteria comprised locally advanced disease (cT3/T4 and/or cN+ stage), and sufficient clinical follow-up data. Patients who received palliative RT, did not complete the prescribed RT course, or had tumors originating from the oral cavity, parotid gland, major/minor salivary glands, external auditory canal/temporal bone, skin of the face/scalp, lateralized sinonasal tumors, nasopharyngeal tumors sites were excluded from the current analysis. Tumors were staged according to the AJCC 8th edition TNM classification. For propensity score matching, pathological T and N stage were used for patients treated with primary surgery followed by adjuvant RT, whereas clinical T and N stage at diagnosis were used for patients treated with definitive RT, reflecting the staging information available for each treatment strategy. HPV-positive oropharyngeal tumors were staged according to the AJCC 8th edition HPV-specific classification.

Clinical information, imaging findings, treatment characteristics, and follow-up data were obtained from institutional electronic medical records. In total, 177 patients in the definitive RT setting and 132 patients in the postoperative setting fulfilled the inclusion criteria and were included in the initial cohort.

All patients underwent CT-based treatment planning using thermoplastic mask immobilization. Gross tumor volume (GTV) included the primary tumor and all radiologically or clinically involved lymph nodes. Clinical target volumes (CTVs) were delineated according to institutional protocols and included a high-risk CTV encompassing the macroscopic tumor, postoperative tumor bed, and involved nodal regions, as well as a low-risk elective nodal CTV. Planning target volumes (PTVs) were generated by adding margins of 3–5 mm to compensate for setup uncertainty and patient motion. Target delineation and dose prescription were performed according to institutional standards independent of HPV status [[9], [10]].

Radiotherapy was administered using volumetric modulated arc therapy (VMAT) in 28–33 fractions. In patients treated with definitive RT, the high-risk clinical target volume (HR-CTV) received a median dose of 70.2 Gy (range: 56–72 Gy), while the low-risk clinical target volume (LR-CTV) received a median dose of 57.6 Gy (range: 50.4–57.6 Gy). In the postoperative setting, median doses of 66 Gy (range: 59.4–67.5 Gy) and 54 Gy (range: 50.4–54 Gy) were prescribed to the HR-CTV and LR-CTV, respectively. Concurrent systemic therapy with platinum-based chemotherapy or cetuximab was administered according to multidisciplinary team recommendations, taking into account tumor characteristics, performance status, and patient comorbidities.

### 2.2 Statistical analysis

To reduce treatment allocation bias and improve comparability between treatment groups of patients receiving surgery followed by adjuvant RT and definitive RT, propensity score matching was performed based on T stage, N stage, and age at diagnosis. These variables were selected because they represented the principal clinical factors influencing treatment allocation within the multidisciplinary tumor board while preserving an adequate matched sample size. HPV status was assessed as a potential matching variable but was not included in the final propensity score model because its distribution was identical between the treatment groups prior to matching with 12/50 patients (24.0%) being HPV-positive and 38/50 (76.0%) HPV-negative in each group (Fisher's exact test, *p* = 1.00).

To minimize treatment selection bias and balance baseline clinicopathologic characteristics between treatment groups, propensity scores were calculated using multivariable logistic regression analysis. One-to-one nearest-neighbor matching without replacement was performed using an absolute propensity-score difference of ≤0.02 (2%) as the caliper.

Balance between matched groups was evaluated using standardized mean differences as well as Fisher's exact tests for categorical variables and the Wilcoxon rank-sum test for continuous variables.

Overall survival (OS) was defined as the time to death from any cause or last follow-up. Progression-free survival (PFS) was defined as the time to the first occurrence of disease progression (local, regional, or distant), death from any cause, or last follow-up. Loco-regional recurrence-free survival (LRFS) was defined as the time to local and/or regional recurrence within the primary tumor site or last follow-up. Patients without an event were censored at the date of last follow-up. Survival outcomes were calculated from the start of radiotherapy using the Kaplan–Meier method, as this represented the common treatment milestone at which oncologic follow-up and toxicity assessment were initiated for both treatment groups. The interval between primary surgery and initiation of postoperative RT was recorded where available; however, complete data were not consistently available for all patients. Differences between treatment groups were assessed using the log-rank test. Hazard ratios (HRs) and 95% CIs were estimated using Cox proportional hazards regression models. Statistical analyses were performed using R statistical software, and all tests were two-sided. A *p*-value <0.05 was considered statistically significant.

Patterns of loco-regional recurrence were categorized based on the methodology by Mohamed et al. [11], as follows: pattern A (central high-dose), pattern B (peripheral high-dose), pattern C (central elective dose), pattern D (peripheral elective dose), and pattern E (extraneous dose). Patients could present with simultaneous recurrences at multiple anatomical sites; therefore, recurrence categories were not mutually exclusive and multiple events could be recorded for a single patient. Recurrences were reviewed by a radiologist and radiation oncologist using follow-up imaging registered to the original planning CT and classified according to the published A–E system; reviewers were not blinded to treatment allocation. Previous malignancy was defined as a documented metachronous non-head-and-neck malignancy diagnosed before the index cancer; no specific time window was applied. Malignancies diagnosed during follow-up were not systematically recorded due to the retrospective study design.

Toxicity related to radiation was assessed retrospectively using clinical documentation and graded according to the Common Terminology Criteria for Adverse Events (CTCAE), version 5.0.

## 3 Results

### 3.1 Matching population

The propensity score–matched cohort consisted of 122 patients, including 61 patients treated with adjuvant RT and 61 patients treated with definitive RT. The distribution of T stage was well balanced between groups, with no statistically significant difference observed between patients treated with adjuvant RT and definitive RT (*p* = 0.696). Similarly, nodal stage distribution was comparable between both cohorts (*p* = 0.923). Age at diagnosis also showed no significant difference after matching. Patients treated with adjuvant RT had a median age of 62 years (mean 62.7 years), whereas patients treated with definitive RT had a median age of 63 years (mean 63.3 years). The Wilcoxon rank-sum test confirmed balanced age distribution between groups (*p* = 0.548). Characteristics of patients before and after matching for the used variables are summarized in Table 1.

**Table 1:** Characteristics of patients before and after matching for the used variables such as T-stage, N-stage and age.

	All patients before Matching							Patients after Matching						
	Adjuvant RT			Definitive RT				Adjuvant RT			Definitive RT			
	N	(%)		n	(%)	*p*-value	SMD	n	(%)		n	(%)	p-value	SMD
Nr. of patients	132			177				61			61			
T-stage														
T1	30	(22.7)		11	(6.2)	<0.001	0.48	6	(9.8)		8	(13.2)	0.696	−0.10
T2	47	(35.6)		37	(20.9)		−0.36	20	(32.8)		19	(31.1)		−0.04
T3	27	(20.5)		64	(36.2)		0.33	15	(24.6)		19	(31.1)		0.14
T4	28	(21.2)		65	(36.7)		0.32	20	(32.8)		15	(24.6)		−0.19
N-Stage														
N0	27	(20.5)		49	(27.7)	<0.001	−0.17	19	(31.1)		15	(24.6)	0.923	0.15
N1	54	(40.99)		24	(13.6)		−0.80	15	(24.6)		16	(26.2)		0.04
N2	31	(23.5)		90	(50.8)		0.55	18	(29.5)		20	(32.8)		0.07
N3	18	(13.6)		12	(6.8)		−0.27	8	(13.2)		8	(13.2)		0.00
Nx	2	(1.5)		2	(1.1)		−0.04	1	(1.6)		2	(3.2)		0.09
Age (years)														
Median	61			67		<0.001		62			63		0.548	
Mean	61.9			67.1			0.56	62.7			63.3			0.07

SMD: standardized mean differences; RT: Radiotherapy

Exact Fisher test (T and N stage), Wilcoxon rank sum test (age)

Baseline characteristics not included in the matching procedure were generally well balanced between treatment groups (see Table 2). Karnofsky performance status, smoking history, alcohol consumption, comorbidities, tumor site, HPV status, and p16 positivity did not significantly differ between patients treated with adjuvant RT and definitive RT. HPV-positive tumors were observed in 30% of the adjuvant RT cohort and 28% of the definitive RT cohort (*p* > 0.999).

**Table 2:** Characteristics of patients treated with adjuvant RT and definitive RT after matching for variables not used for matching.

*Variable*	***Adjuvant RT*** ***(n*** ***=*** ***61; 100%)***	***Definitive RT (n*** ***=*** ***61; 100%)***	*p-value*	*SMD after Matching*
**Karnofsky-Index**				0.18
- 90–100%	30 (49%)	33 (54%)	0.717	
- 80–70%	30 (49%)	28 (46%)		
- 60%	1 (2%)	0 (0%)		
**Smoke**				0.17
- Never	6 (14%)	7 (15%)	**0.677**	
- Quit	11 (25%)	8 (17%)		
- Current	27 (61%)	31 (67%)		
- Missing	17	19		
**Alcohol**				0.39
- yes	25 (71%)	19 (53%)	0.144	
- no	10 (29%)	17 (47%)		
- missing	26	25		
**Comorbidity**				
- COPD	2 (3%)	4 (7%)	0.680	0.17
- Other lung disease	1 (2%)	3 (5%)	**0.619**	0.26
- Heart disease	11 (18%)	11 (18%)	>0.999	0.00
- Arterial hypertension	32 (53%)	25 (41%)	0.276	0.23
- Diabetes	8 (13%)	3 (5%)	0.205	0.31
- Other malignancies	2 (3%)	14 (23%)	0.002	0.63
- Stroke	4 (7%)	4 (7%)	>0.999	0.00
**Tumor site**				0.28
- oropharynx	34 (56%)	39 (64%)	0.381	
- hypopharynx	8 (13%)	10 (16%)		
- larynx	19 (31%)	12 (20%)		
**HPV-status**				0.04
- positive	18 (30%)	17 (28%)	>0.999	
- negative	43 (70%)	44 (72%)		
**p-16 positive**	16 (26%)	8 (13%)	0.110	0.34
**Concurrent systemic therapy**				1.04
Cisplatin alone	23 (38%)	34 (56%)	0.069	0.37
Cetuximab	0 (0%)	5 (8%)	0.057	0.42

SMD: standardized mean differences; HPV: human papilloma virus; RT: radiotherapy.

Most comorbidities were similarly distributed between groups; however, previous malignancies were significantly more frequent in patients treated with definitive RT compared with adjuvant RT (23% vs. 3%, *p* = 0.002). Concurrent systemic therapy tended to be administered more often in the definitive RT group, with higher rates of cisplatin-based chemotherapy (56% vs. 38%, *p* = 0.069) and cetuximab use (8% vs. 0%, *p* = 0.057), although these differences did not reach statistical significance.

### 3.2 Toxicity profile related to radiation after matching

Radiation treatment-related toxicities were generally comparable between patients treated with adjuvant RT and definitive RT (Table 3). The most common toxicities in both groups included fatigue, dysphagia, taste disorders, xerostomia, mucositis, and dermatitis. No significant differences were observed regarding fatigue (64% vs. 77%, *p* = 0.164), dysphagia (74% vs. 84%, *p* = 0.269), xerostomia (59% vs. 69%, *p* = 0.346), mucositis (59% vs. 74%, *p* = 0.125), or dermatitis of any grade (90% in both groups, *p* > 0.999). Rates of severe toxicities, including grade ≥ 3 weight loss and dermatitis, were low and comparable between cohorts.

**Table 3:** Comparison of Treatment-Related Toxicities Between Patients treated with adjuvant RT vs definitive RT after Matching.

	
**Toxicity**	**Adjuvant RT**	**Definitive RT**	***p-value***
	**N %**	**N %**	
Fatigue	39 (64%)	47 (77%)	0.164
Weight loss - all grades - grade ≥ 3	35 (57%) 3 (5%)	37 (61%) 3 (5%)	0.854 >0.999
Dysphagia	45 (74%)	51 (84%)	0.269
Taste disorders	41 (67%)	45 (74%)	0.552
Xerostomia	36 (59%)	42 (69%)	0.346
Mucositis	36 (59%)	45 (74%)	0.125
Dermatitis - all grades - grade ≥ 3	55 (90%) 6 (10%)	55 (90%) 2 (3%)	**>0.999** **0.272**
Skin hyperpigmentation	12 (20%)	19 (31%)	0.212
Lymphedema	20 (33%)	8 (13%)	*0.017*
Hearing loss	2 (3%)	0 (0%)	0.496
Osteoradionecrosis	2 (3%)	1 (2%)	>0.999
PEG after RT	3 (5%)	2 (3%)	>0.999

PEG: percutaneous endoscopic gastrostomy, RT: radiotherapy.

Lymphedema occurred more frequently in patients treated with primary surgery followed by adjuvant RT than in those treated with definitive RT (33% vs. 13%, *p* = 0.017). No significant differences were observed regarding hearing loss, osteoradionecrosis, or long-term percutaneous endoscopic gastrostomy (PEG) dependence after RT.

### 3.3 Outcome after propensity score matching

The median follow-up was 29.0 months (range: 12.0–64.2 months) in the adjuvant RT cohort and 23.4 months (range: 12.6–40.8 months) in the definitive RT cohort. Mean follow-up durations were 37.8 ± 28.9 months and 27.6 ± 18.7 months, respectively.

The median OS was 81 months in the adjuvant RT cohort, whereas the median OS was not reached in the definitive RT cohort. Five-year OS rates were 63.9% (95% CI, 50.4–80.9%) and 60.0% (95% CI, 44.9–80.2%), respectively. Consistent with this finding, Kaplan–Meier analysis showed no significant difference in OS between groups (HR 1.05; 95% CI, 0.50–2.22; log-rank *p* = 0.70) (Fig. 1).

**Fig. 1:**
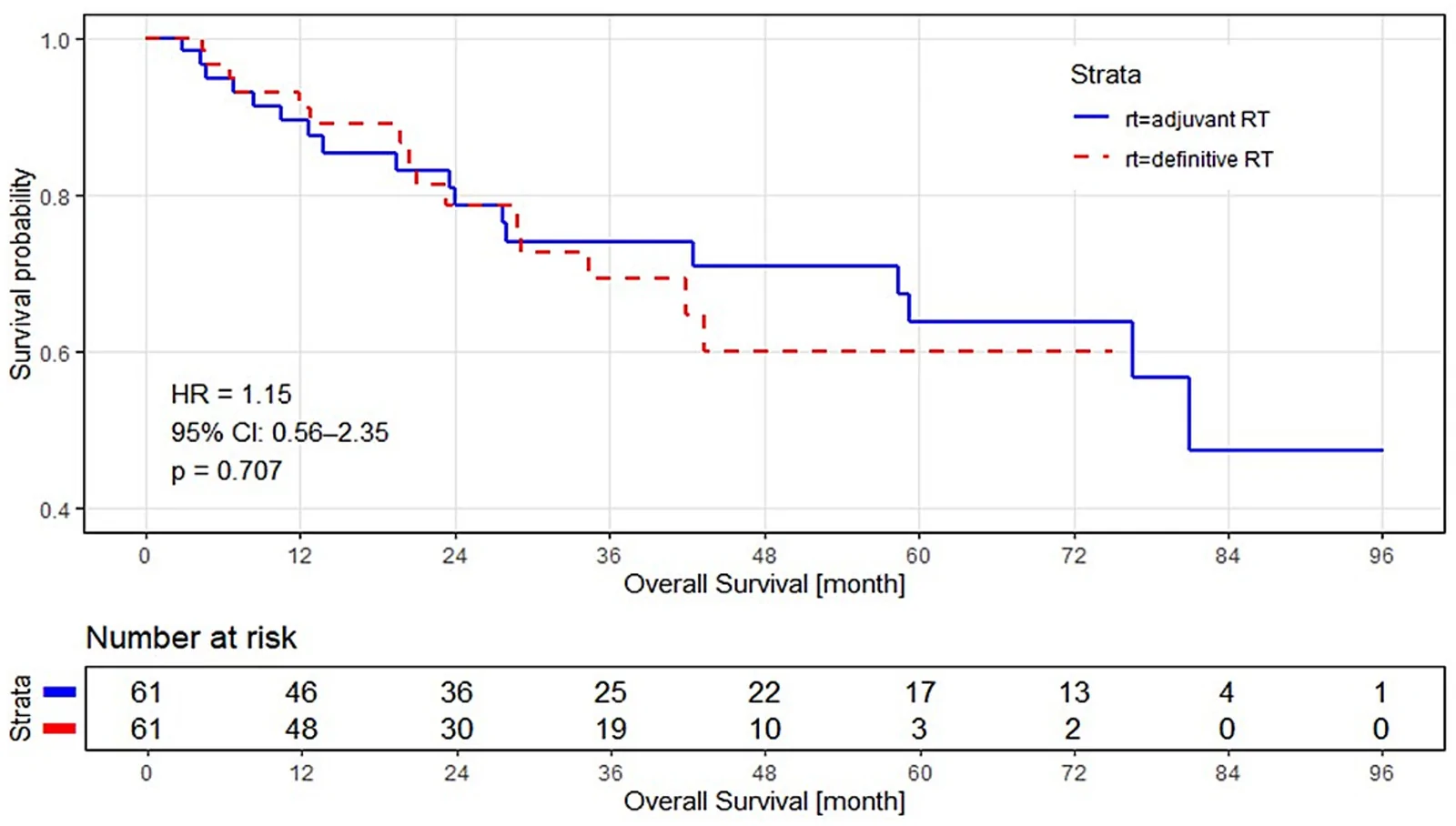
overall survival between groups: adjuvant RT and definitive RT (*p* = 0.70).

The median PFS was 81.0 months after adjuvant RT compared with 43.4 months after definitive RT. Five-year PFS rates were 58.7% (95% CI, 45.1–76.4%) and 48.1% (95% CI, 34.1–67.7%), respectively. Kaplan–Meier analysis showed no statistically significant difference in PFS between the treatment groups, although numerical differences favored adjuvant RT (HR 1.70, 95% CI 0.92–3.13; log-rank *p* = 0.092) (Fig. 2). Median LRFS was not reached after adjuvant RT, whereas it was 61.5 months after definitive RT. Five-year LRFS rates were 85.8% (95% CI, 76.5–96.2%) and 74.6% (95% CI, 63.3–87.7%), respectively. Kaplan–Meier analysis demonstrated numerical differences favored loco-regional control after adjuvant RT (HR 2.25; 95% CI, 0.89–5.65; log-rank *p* = 0.085) (Fig. 3).

**Fig. 2:**
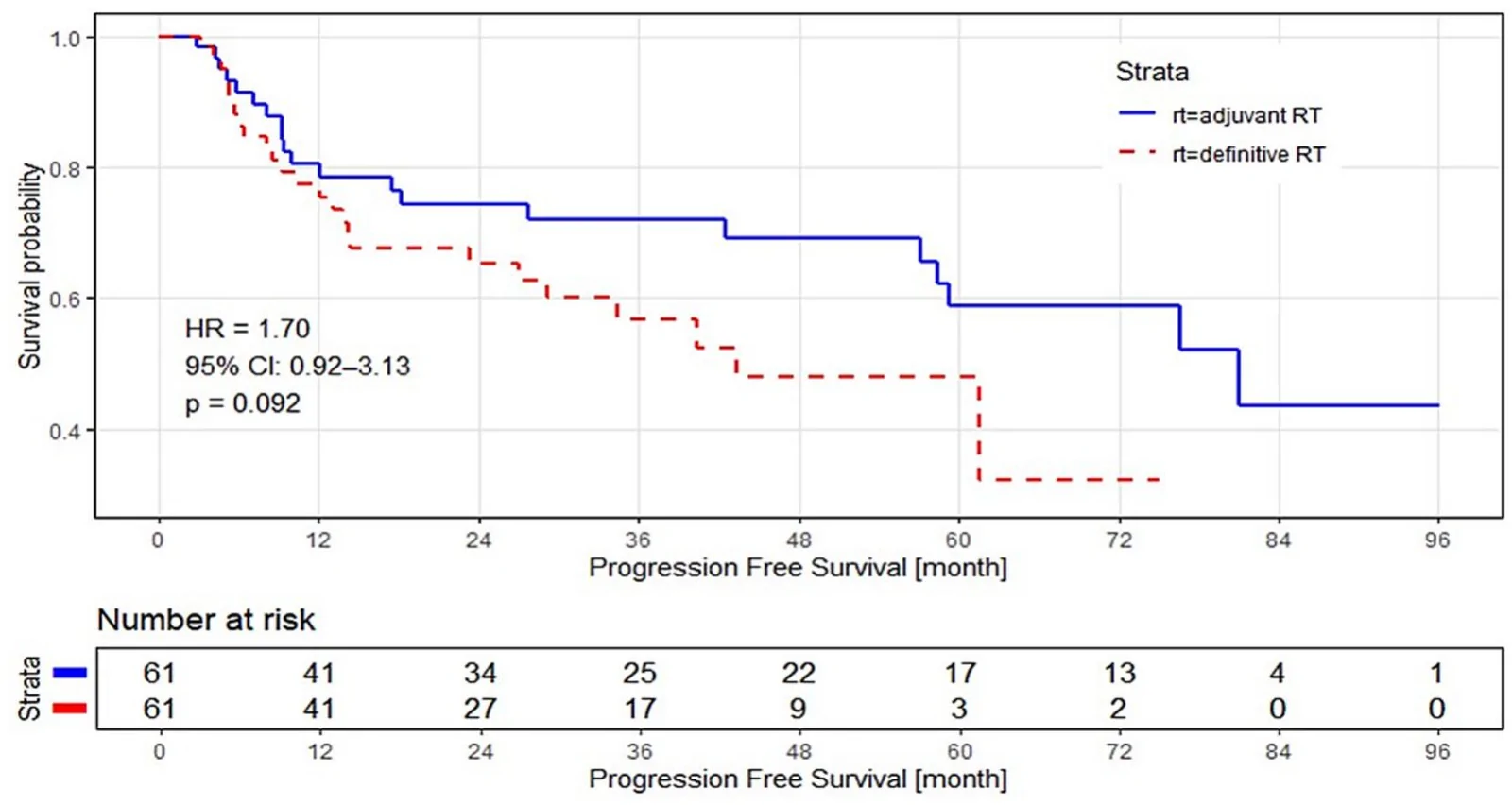
progression free survival between groups: adjuvant RT and definitive RT (*p* = 0.09).

**Fig. 3:**
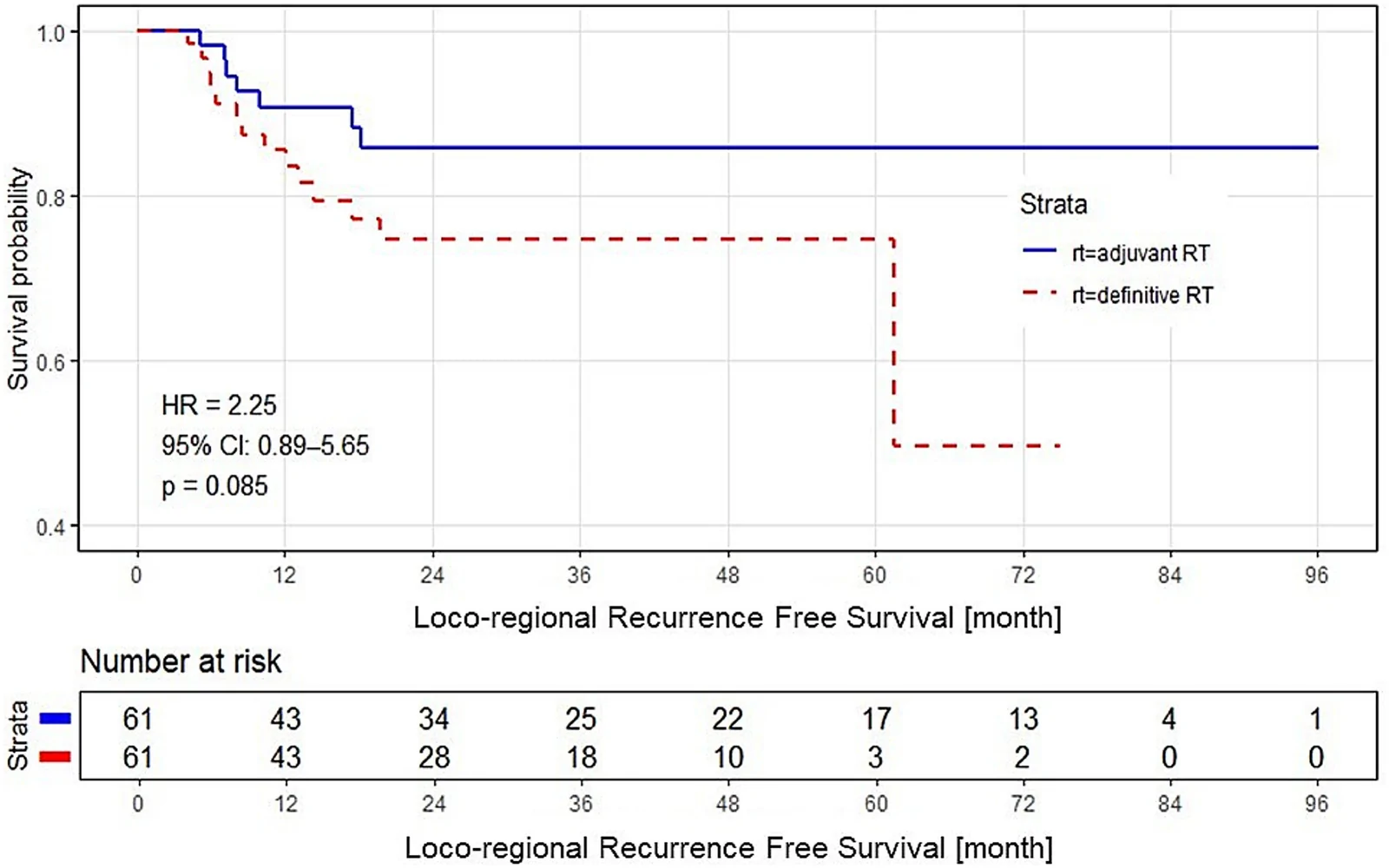
loco-regional recurrence free survival between groups: adjuvant RT and definitive RT (*p* = 0.08).

Because a history of previous malignancy remained imbalanced after matching, additional multivariable Cox regression analyses were performed. Previous malignancy was not independently associated with overall survival (HR 1.71, 95% CI 0.63–4.61, *p* = 0.290), progression-free survival (HR 1.54, 95% CI 0.67–3.55, *p* = 0.309), or loco-regional recurrence-free survival (HR 2.09, 95% CI 0.73–5.98, *p* = 0.167) (supplementary tables 1a-c).

### 3.4 Loco-regional recurrence patterns after matching

Loco-regional recurrence occurred less frequently after adjuvant RT than after definitive RT (12% vs. 23%), although this difference was not statistically significant (*p* = 0.149) (see Table 4). Because some patients experienced simultaneous recurrences at multiple locations, recurrence categories were not mutually exclusive (multiple entries possible per patient). In both groups, most recurrences developed within the central high-dose radiation volume. Central recurrences of the primary tumor site were observed in 43% of recurrences after adjuvant RT and 64% after definitive RT. Nodal recurrences within the high-dose region were also more common following definitive RT. Marginal and out-of-field recurrences were uncommon in both cohorts. Overall, most treatment failures occurred within previously irradiated target volumes.

**Table 4:** Patterns of Local and Regional Recurrence in Patients treated with adjuvant RT and definitive RT after Matching.

**Patterns of relapse [11] overall *n*** **=** **21**			
Adjuvant RT *N* = 7/61 (12%)		Definitive RT *n* = 14/61 (23%)	p-value 0.149
***Group A (central high dose volume)***			
- *1 (primary tumor)*	3/7 (43%)	9/14 (64%)	0.397
- *2 (positive nodes)*	1/7 (14%)	6/13 (46%)	0.329
***Group B (marginal high dose volume)***			
- *1 (primary tumor)*	1/7 (14%)	3/14 (21%)	>0.999
- *2 (positive nodes)*	2/7 (29%)	3/14 (21%)	>0.999
***Group C (central elective volume)***	4/7 (57%)	3/14 (21%)	0.156
***Group D (marginal elective volume)***	0/7 (0%)	3/14 (21%)	0.521
***Group E (outside of RT volume in head and neck region)***	2/7 (29%)	3/14 (21%)	>0.999

## 4 Discussion

In this propensity score-matched study, we compared two standard curative treatment strategies such as primary surgery followed by adjuvant RT and definitive RT for patients with locally advanced squamous cell carcinoma of the pharynx and larynx.

Locally advanced HNSCC is commonly treated either with definitive radiotherapy-based organ preservation strategies or surgery followed by adjuvant RT with or without systemic therapy. Modern multimodal treatment approaches have resulted in 5-year overall survival rates of approximately 50–70% and loco-regional control rates ranging from 65 to 85%, depending on tumor site, stage, HPV status, and treatment modality [12]. Several retrospective large studies have demonstrated comparable overall survival between definitive chemoradiation and surgery-based approaches, while postoperative RT has frequently shown improved loco-regional control, particularly in patients with high-risk pathological features [13], [14].

Several established prognostic factors are known to negatively influence survival and loco-regional control in locally advanced HNSCC. In particular, extracapsular extension (ECE), positive surgical margins, advanced nodal disease (pN2/3), and advanced primary tumor stage (pT3/4) have repeatedly been associated with inferior oncologic outcomes and increased recurrence risk. The landmark trials by Cooper et al. and Bernier et al. demonstrated that patients with ECE or microscopically involved margins significantly benefit from treatment intensification with concurrent chemoradiotherapy in the postoperative setting [15], [16]. Similarly, more recent propensity score analyses confirmed poorer outcomes in patients with advanced nodal and tumor stage despite multimodal therapy [17].

The current propensity score–matched analysis demonstrated comparable OS outcomes between patients with locally advanced SCC of the pharynx and larynx treated with primary surgery followed by adjuvant RT.

Since the treatment period of the present cohort, perioperative and postoperative immune checkpoint inhibition has emerged as a practice-changing component in resectable locally advanced HNSCC. In KEYNOTE-689, the addition of neoadjuvant and adjuvant pembrolizumab to surgery and risk-adapted adjuvant radiotherapy or chemoradiotherapy improved event-free survival compared with standard care [18]. Similarly, NIVOPOSTOP demonstrated improved disease-free survival when nivolumab was added to postoperative cisplatin-based chemoradiotherapy in patients with resected high-risk locally advanced SCCHN [19]. Therefore, the present analysis should be interpreted as a contemporary real-world benchmark of radiotherapy-based treatment strategies before broad implementation of perioperative PD-1 blockade.

However, numerical differences in PFS and LRFS favored patients treated with surgery followed by adjuvant RT, although these differences were not statistically significant. Similar findings have been reported in previous retrospective and propensity score–matched studies in head and neck cancer patients. Rudat et al. compared definitive and postoperative radiotherapy in oro- and hypopharyngeal cancer and likewise observed comparable survival outcomes between both treatment approaches, emphasizing the importance of tumor-related prognostic factors such as the total tumor volume rather than treatment modality alone [14].

The absence of a significant difference in overall survival in our study suggests that both treatment strategies remain effective approaches for the management of locally advanced head and neck cancer. Definitive RT continues to represent an important organ-preserving treatment option, particularly in patients with extensive local disease or increased surgical risk. At the same time, the numerically improved disease control observed after adjuvant RT may reflect surgical cytoreduction, pathological risk selection, residual confounding, or differences in tumor burden. In a recent propensity score–matched analysis by Liu et al., postoperative chemoradiotherapy demonstrated improved loco-regional relapse-free survival compared to postoperative RT alone in patients with pT3–4 or pN2–3 disease, highlighting the relevance of aggressive local treatment in selected high-risk patients, even though toxicity rates were higher in patients receiving postoperative chemoradiotherapy [17].

Loco-regional recurrence was numerically less frequent after primary surgery followed by adjuvant RT than after definitive RT (12% vs. 23%), although this difference was not statistically significant (*p* = 0.149). Given the limited sample size, this finding should be considered exploratory and hypothesis-generating.

Most recurrences occurred within previously irradiated high-dose target volumes, which is consistent with patterns of failure reported in modern RT series [2]. Local disease control remains a major therapeutic objective in head and neck oncology because loco-regional recurrence is frequently associated with substantial morbidity, impaired swallowing and speech function, and poor salvageability [20]. Previous matched analyses in head and neck SCC similarly demonstrated that improved loco-regional control may translate into favorable long-term outcomes, even in the absence of significant overall survival differences [21], [22].

Observational studies in head and neck oncology are particularly susceptible to treatment allocation bias because patients selected for definitive RT are often older, medically frailer, or present with more advanced disease [23]. This is particularly relevant in older or medically vulnerable patients, as recent multicenter work by Marschner et al. demonstrated that routinely available clinical variables can meaningfully stratify survival outcomes in older adults with HNSCC undergoing chemoradiation [24]. Propensity score methods have therefore become increasingly established for comparative effectiveness analyses in HNSCC and have been successfully applied in several recent studies evaluating postoperative RT, chemoradiotherapy, and definitive RT strategies (17, 22,25).

An important strength of this study is the use of propensity score matching to reduce baseline confounding. Prior to matching, major differences existed regarding tumor stage and patient age, both of which significantly influenced treatment allocation. After matching, balance was substantially improved for the prespecified matching variables; however, residual imbalances remained in several clinically relevant characteristics such as previous malignancy and systemic therapy.

Nevertheless, several limitations should be acknowledged. The retrospective single-center design inherently carries the risk of residual confounding and selection bias. The relatively limited matched sample size reduced statistical power and may have contributed to the inability to demonstrate statistically significant differences in progression-free and loco-regional recurrence-free survival despite numerical trends. Survival outcomes were calculated from the start of radiotherapy, which may have introduced immortal time/selction bias in the adjuvant RT cohort. The relatively short median follow-up also limits the reliability of the 5-year Kaplan–Meier estimates, which should therefore be interpreted with caution. Differences in concurrent systemic therapy, particularly the use of cisplatin and cetuximab, may have contributed to the observed oncologic outcomes and should therefore be considered when interpreting the results. Another limitation is the use of pathological TNM staging in the adjuvant RT cohort versus clinical staging in the definitive RT cohort, which may limit comparability despite propensity score matching. Furthermore, key postoperative risk factors, including margin status, extranodal extension, nodal burden, and lymphovascular or perineural invasion, were incompletely available. Thus, residual confounding by postoperative disease risk and treatment indication cannot be excluded. Despite propensity score matching, previous malignancies remained significantly more frequent in the definitive RT cohort (23% vs. 3%, *p* = 0.002). This imbalance likely reflects real-world treatment selection, as patients with a history of malignancy are often considered less suitable candidates for extensive surgery and are therefore preferentially treated with definitive radiotherapy. Although adjustment for previous malignancies did not materially alter the survival analyses, residual confounding cannot be excluded and represents an inherent limitation of this retrospective study. The five-year survival estimates should be interpreted with caution, particularly in the definitive RT group, because only a small number of patients remained at risk at 60 months.

Future prospective studies are needed to further define which patient subgroups may benefit most from either treatment strategy and to optimize individualized treatment selection in locally advanced head and neck cancer.

## 5 Conclusions

Primary surgery followed by adjuvant RT and definitive RT achieved comparable overall survival after propensity score matching. Although surgery followed by adjuvant RT demonstrated trends toward improved progression-free and loco-regional recurrence-free survival, these differences were not statistically significant. Treatment decisions should therefore remain individualized within a multidisciplinary setting. These findings support the need for prospective validation in larger patient cohorts.

## Permission notes

We did not use any material in the manuscript that is not original content.

## CRediT authorship contribution statement

**Linda Agolli:** Writing – original draft, Visualization, Project administration, Methodology, Formal analysis, Conceptualization. **Elidon Mici:** Writing – original draft, Visualization, Software, Methodology, Investigation, Formal analysis, Data curation, Conceptualization. **Christine Langer:** Writing – review & editing, Validation, Supervision, Data curation. **Gabriele A. Krombach:** Writing – review & editing, Validation, Supervision, Resources. **Sebastian Harth:** Writing – review & editing, Supervision, Software, Resources. **Leon Wendrich:** Software, Resources, Project administration, Formal analysis, Data curation. **Stefan Gattenlöhner:** Writing – review & editing, Validation, Resources. **Christoph Arens:** Writing – review & editing, Validation, Supervision, Methodology, Investigation, Conceptualization. **Daniel Habermehl:** Writing – review & editing, Supervision, Methodology, Conceptualization.

## Ethics declaration

The authors have NOT obtained informed consent from participants or their legal representatives. Waived by the Ethics Committee due to the retrospective nature of the study.

This study was performed in compliance with relevant laws, regulatory frameworks and guidelines where the research took place.

This study was approved by the Justus-Liebig University of Giessen, University Hospital Giessen, Germany.

(Approval No. AZ 73/24)

## Ethics

The study was performed in accordance with the Declaration of Helsinki Version 2013. The current study was approved from our internal review board of Justus-Liebig University of Giessen (ethic approval AZ 73/24 – Ethik-Kommission des Fachbereiches Medizin).

## Human Ethics and Consent to Participate declarations

Waived by the Ethics Committee due to the retrospective nature of the study.

## Declaration of competing interest

The authors declare that they have no known competing financial interests or personal relationships that could have appeared to influence the work reported in this paper.
