# Supplementary material for: Surgery followed by adjuvant radiotherapy versus definitive radiotherapy for locally advanced squamous cell carcinoma of the pharynx and larynx: a propensity score-matched analysis
Source: Clin Transl Radiat Oncol. 2026 Sep 10;62:101288. doi: 10.1016/j.ctro.2026.101288 (PMC13631610; doi:10.1016/j.ctro.2026.101288)
Supplement: Supplementary file 1 [file mmc1.docx]

**Supplementary Table 1a. Cox proportional hazards analysis for overall survival (OS)**

| Variable | Regression coefficient (B) | Standard Error | Hazard Ratio (HR) | 95% CI | p-value |
| --- | --- | --- | --- | --- | --- |
| Definitive RT vs. adjuvant RT | 0.051 | 0.380 | 1.15 | 0.56–2.35 | 0.707 |
| Previous malignancy (yes vs. no) | 0.536 | 0.506 | 1.709 | 0.63–4.61 | 0.290 |

**Supplementary Table 1b. Cox proportional hazards analysis for progression-free survival (PFS)**

| Variable | Regression coefficient (B) | Standard Error | Hazard Ratio (HR) | 95% CI | p-value |
| --- | --- | --- | --- | --- | --- |
| Definitive RT vs. adjuvant RT | 0.465 | 0.323 | 1.70 | 0.92–3.13 | 0.092 |
| Previous malignancy (yes vs. no) | 0.433 | 0.425 | 1.541 | 0.67–3.55 | 0.309 |

**Supplementary Table 1c. Cox proportional hazards analysis for local recurrence-free survival (LRFS)**

| Variable | Regression coefficient (B) | Standard Error | Hazard Ratio (HR) | 95% CI | p-value |
| --- | --- | --- | --- | --- | --- |
| Definitive RT vs. adjuvant RT | 0.665 | 0.490 | 2.25 | 0.89–5.65 | 0.085 |
| Previous malignancy (yes vs. no) | 0.738 | 0.535 | 2.092 | 0.73–5.98 | 0.167 |

**Abbreviations:** RT, radiotherapy; HR, hazard ratio; CI, confidence interval; LRFS, local recurrence-free survival; PFS, progression-free survival; OS, overall survival.
